# Molecular evidence of sustained urban malaria transmission in Amazonian Brazil, 2014–2015

**DOI:** 10.1017/S0950268820000515

**Published:** 2020-02-21

**Authors:** L. C. Salla, P. T. Rodrigues, R. M. Corder, I. C. Johansen, S. Ladeia-Andrade, M. U. Ferreira

**Affiliations:** 1 Department of Parasitology, University of São Paulo, São Paulo, Brazil; 2 Laboratory of Parasitic Diseases, Oswaldo Cruz Foundation, Rio de Janeiro, Brazil

**Keywords:** Amazon, malaria, molecular epidemiology, population structure, urban

## Abstract

The relative contribution of imported *vs.* locally acquired infections to urban malaria burden remains largely unexplored in Latin America, the most urbanised region in the developing world. Here we use a simple molecular epidemiology framework to examine the transmission dynamics of *Plasmodium vivax* in Mâncio Lima, the Amazonian municipality with the highest malaria incidence rate in Brazil. We prospectively genotyped 177 *P. vivax* infections diagnosed in urban residents between June 2014 and July 2015 and showed that local parasites are structured into several lineages of closely related microsatellite haplotypes, with the largest genetic cluster comprising 32% of all infections. These findings are very unlikely under the hypothesis of multiple independent imports of parasite strains from the rural surroundings. Instead, the presence of an endemic near-clonal parasite lineage circulating over 13 consecutive months is consistent with a local *P. vivax* transmission chain in the town, with major implications for malaria elimination efforts in this and similar urban environments across the Amazon.

## Introduction

The epidemiology of urban malaria remains little explored in Latin America, the most urbanised region in the developing world [1]. Malaria rates tend to be lower in cities and towns compared with neighbouring rural settings, due to multiple factors such as improved housing and access to healthcare and limited availability of suitable vector-breeding habitats [2]. Nevertheless, malaria cases have been increasingly reported within and near urban centres across the Amazon Basin [3–7] and the Pacific Coast of South America [8–13].

Urban malaria is affected by human mobility patterns, as parasites from the rural communities of high endemicity may be introduced into urbanised spaces and may be locally transmitted, leading to micro-epidemics or sustained endemic propagation in cities and towns [9, 10, 14]. Unsurprisingly, routine surveillance often counts imported malaria infections that are diagnosed in urban health facilities as ‘urban cases’ [2, 12], even when there is no evidence of transmission in or around urban dwellings, as required to properly characterise ‘urban malaria transmission’ [2].

Brazil has experienced a dramatic decline in malaria incidence over the past three decades, but residual transmission remains entrenched in the Amazon Basin [15]. This region, which contributes over 99% of the country's malaria burden [15], has 72.6% of its population presently living in urbanised settlements that range from large cities to small towns sprawling into the nearby forest [16]. Nearly 90% of the malaria burden countrywide is nowadays due to *Plasmodium vivax* [17].

**Fig. 1.**
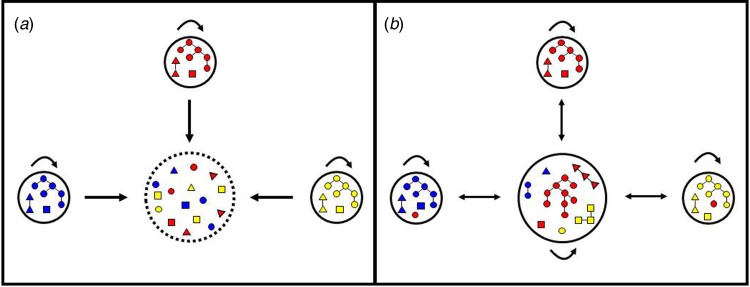
Hypotheses regarding the origin of urban malaria cases. (a) Cases diagnosed in urban residents are exclusively imported from rural villages. In this scenario, the urban centre is purely a sink (represented by interrupted lines) and the rural villages are the sources of parasites introduced into the urban centre, but are not connected to each other. Arrows indicate parasite mobility between locations and self-loops indicate parasite transmission within each site. Note that colour-coded parasite lineages from each of the three villages (represented by small circles) are introduced into the urban centre (large circle) but are not further transmitted in the town, as indicated by the absence of a self-loop. Each parasite lineage found in urban dwellers has been independently introduced by migration; identical or closely related parasites are occasionally found in the town because different people may have been infected in the same source village. (b) Cases diagnosed in urban residents may be either imported or locally acquired. In this scenario, parasite lineages from rural villages are introduced into the urban area and further spread locally among urban residents, as indicated by the presence of a self-loop. As a result, parasite transmission chains (represented as parasites connected by lines) are observed in urban residents. Parasites in the same transmission chain form clusters of identical or closely related haplotypes (clonal or near-clonal lineages) here termed haplogroups. Human mobility also introduces into rural villages some parasite lineages that originate from another rural village but circulate in the town; therefore, the town is both a source and a sink of parasites.

Here we introduce a simple molecular epidemiology framework (Fig. 1) to explore the relative contribution of urban transmission chains to residual malaria in the Juruá Valley region, the hotspot in north-western Brazil that contributes 18% of the country's malaria burden [17]. We use a simple metrics – the observed distance between each haplotype and the nearest haplotype found in the population, here termed ‘distance to nearest' – to identify clustering patterns of local parasites and test whether they deviate from panmictic expectations. We show that nearly one-third of parasites from urban residents who were infected over 14 months of follow-up clustered into a single endemic near-clonal *P. vivax* lineage, consistent with sustained local malaria transmission.

## Methods

### Study site

**Fig. 2.**
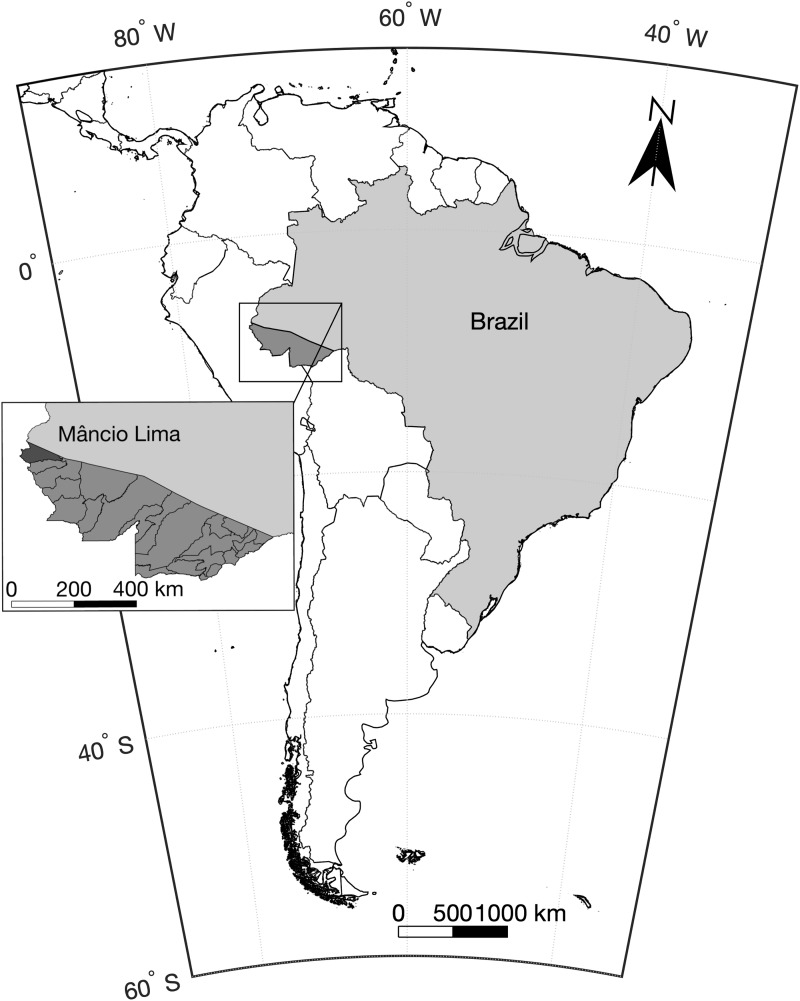
Map of South America showing the location of the study site, the municipality of Mâncio Lima in the state of Acre, north-western Brazil, next to the border with Peru.

This study was carried out in the municipality or county of Mâncio Lima, in upper Juruá Valley, next to the Brazil–Peru border, where 45% of all malaria cases are reportedly acquired in urban settings, compared with the country's average of 15% [18]. Mâncio Lima covers a surface area of 5453 km^2^ in north-western Brazil (Fig. 2) and comprises a single town (07°36′51″S, 72°53′45″W), where half of its 18 638 inhabitants reside. Streams, wetlands rich in moriche palm trees, and man-made fish-farming ponds are widespread across the town. With a typical equatorial humid climate, Mâncio Lima receives most rainfall between November and April, but malaria transmission occurs year-round. The annual parasite incidence, estimated at 521 cases per 1000 inhabitants in 2017, is the highest for a municipality in Brazil [17], but only households perceived to be at increased risk are routinely targeted for long-lasting insecticidal bed-net distribution and indoor residual spraying with pyrethroids. The primary local malaria vector is *Anopheles darlingi*, but *An. albitarsis s.l.* is also frequently found in natural and man-made larval habitats widely distributed across the town, especially in fish-farming ponds [19, 20].

### Study population and parasite DNA samples

A *P. vivax* isolate was defined as a sample of parasites derived from a single patient at a single occasion; one isolate may comprise one or more genetically diverse clones. We prospectively sampled *P. vivax* isolates, from June 2014 through July 2015, from 178 residents in the town of Mâncio Lima aged between 5 and 63 years and participating in an open-label randomised clinical trial to monitor chloroquine–primaquine efficacy for vivax malaria treatment [21]. Eligible subjects were symptomatic patients of either sex, with age between 5 and 70 years, attending government-run clinics in the town of Mâncio Lima with a *P. vivax* infection confirmed by microscopy and polymerase chain reaction (PCR); no minimal parasite density threshold was set. Only urban residents were enrolled because directly observed therapy and post-treatment follow-up would be impractical in remote rural sites. Exclusion criteria were severe or complicated malaria, pregnancy or lactation, severe malnutrition, severe anaemia and other serious chronic clinical conditions, glucose-6-phosphate dehydrogenase deficiency, antimalarial use in the preceding 2 weeks and known hypersensitivity or allergy to study drugs. Spatial analyses considered only subjects whose houses had GPS coordinates determined. We used 200-μl of anticoagulated blood drawn before treatment to isolate parasite's DNA, using QIAamp DNA blood kits (Qiagen, Hilden, Germany). A single pre-treatment sample was analysed for each participant.

### Parasite genotyping

We typed 14 single-copy microsatellite loci that map to 10 different chromosomes (Table 1); 13 of them consist of trinucleotide repeats and one of tetranucleotide repeats [22]. Microsatellite alleles were PCR amplified exactly as previously described [23] and PCR products were separated by capillary electrophoresis on an automated DNA sequencer ABI 3730 (Applied Biosystems, Foster City, CA). The lengths (in base pairs (bp)) and relative abundance (peak heights in electropherograms) of the PCR-amplified alleles were determined using GeneMarker 2.7 (Soft Genetics LLC, State College, PA) software. The minimal detectable peak height was set to 900 arbitrary fluorescence units and we scored two alleles at a locus when the minor peak was >33% the height of the predominant peak. Samples were considered to contain multiple genetically distinct subpopulations if at least one locus showed more than one allele. Haplotypes were defined as unique combinations of alleles at each locus analysed, considering only the most abundant alleles for haplotype assignment in multiple-clone infections [23].

**Table 1.** Overall diversity of 14 microsatellite loci in 177 *P. vivax* isolates from the town of Mâncio Lima, Brazil (2014–15)

Locus	Chromosome	Allele size range^a^	No. of alleles (*k*)	*H*_E_^b^
MS1	3	228–246	6	0.57
MS2	6	194–238	9	0.49
MS3	4	173–197	7	0.62
MS4	6	183–210	7	0.60
MS5	6	160–229	8	0.74
MS6	11	192–258	13	0.69
MS7	12	139–154	3	0.26
MS8	12	207–300	19	0.81
MS9	8	146–176	9	0.74
MS10	13	183–258	13	0.79
MS12	5	197–266	11	0.73
MS15	5	236–311	13	0.79
MS16	9	267–357	15	0.77
MS20	10	195–258	11	0.72

a Amplicon size in base pairs.

b *H*_E_ = expected heterozygosity, defined as 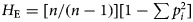, where *n* is the number of isolates analysed and *p*_*i*_ is the frequency of the *i*-th allele in the population.

### Data analysis

We used Arlequin 3.5 software [24] to calculate the expected heterozygosity (*H*_E_) as a measure of overall genetic diversity, defined as 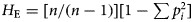, where *n* is the number of isolates analysed and *p*_*i*_ is the frequency of the *i*-th allele in the population. *H*_E_ gives the average probability that a pair of alleles randomly obtained from the population is different and ranges between 0 and 1. We next calculated the pairwise number of different alleles or mismatches, out of 14 loci analysed, as an estimate of genetic distance between isolates. The observed mismatch distribution was compared with that expected under random association of alleles in a panmictic population, which was generated by using 50 000 simulated datasets in which alleles were randomly reshuffled among haplotypes using the *randperm* function of MATLAB (MathWorks, Natick, MA). Finally, we calculated the distance between each haplotype and the nearest haplotype found in the population, here termed ‘distance to nearest', which ranges from 0 (identical haplotypes) to 14 mismatches (different alleles at all loci typed). Similarly, we estimated the distance-to-nearest distribution that is expected under panmixia by randomly reshuffling alleles among haplotypes as above.

The standardised index of association 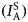 was used as a measure of overall multi-locus linkage disequilibrium (LD) in the parasite population. 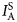 compares the observed variance (*V*_D_) of the number of alleles at which each pair of haplotypes differ in the population (the mismatch distribution as above) with the variance expected under random association of alleles (*V*_E_) as follows: 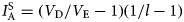, where *l* is the number of loci analysed [25]. *V*_E_ was derived from 10 000 random permutations. LD is expected to increase *V*_D_ relative to *V*_E_ [25]; significant LD is detected whenever *V*_D_ was greater than 95% of *V*_E_ values. Data were analysed with LIAN 3.5 software [26].

We used the goeBURST algorithm [27] implemented in PHYLOViZ 2.0 software [28], which clusters samples having at least one connection above a given similarity threshold, to assign haplotypes to haplogroups and represent haplotype genealogies as minimum spanning trees. We define haplogroups as clusters of related haplotypes that share at least 12 alleles with one or more haplotypes within the same cluster. The goeBURST algorithm generates haplotype genealogies by assuming that a founder haplotype propagates in the population and gradually diversify, as a result of mutation or recombination, to originate a cluster of genetically related haplotypes or lineage that can still be traced to their ancestral haplotype [27].

This study follows the STROBE guidelines for observational studies and its extension STROME-ID for molecular epidemiological studies.

### Ethics statement

The authors assert that all procedures contributing to this work comply with the ethical standards of the relevant national and institutional committees on human experimentation and with the Helsinki Declaration of 1975, as revised in 2008. Study protocols have been approved by the Institutional Review Board of the University Hospital of the University of São Paulo (550.565). Written informed consent was obtained from all study participants or their parents/guardians.

## Results

### Clonal structure in an urban *Plasmodium vivax* population

Between June 2014 and July 2015, 1487 *P. vivax* infections were laboratory confirmed among urban residents in Mâncio Lima. DNA samples from 177 of them were completely genotyped at all 14 microsatellite loci (11.9% of all infections diagnosed in urban residents during the study period and 99.4% of the available samples). Further analyses only considered fully typed isolates. The temporal distribution of genotyped *P. vivax* infections followed closely that of all *P. vivax* infections diagnosed in Mâncio Lima throughout the study period (Supplementary Fig. S1). The urban population of *P. vivax* was nearly as diverse (average *H*_E_, 0.66; s.d. of 0.15) as its rural counterparts across the Brazilian Amazon (*H*_E_ ranging from 0.68 to 0.74) [29]. Overall, 45.2% of isolates comprised multiple clones and 16.4% had two alleles scored at ≥2 different loci.

**Fig. 3.**
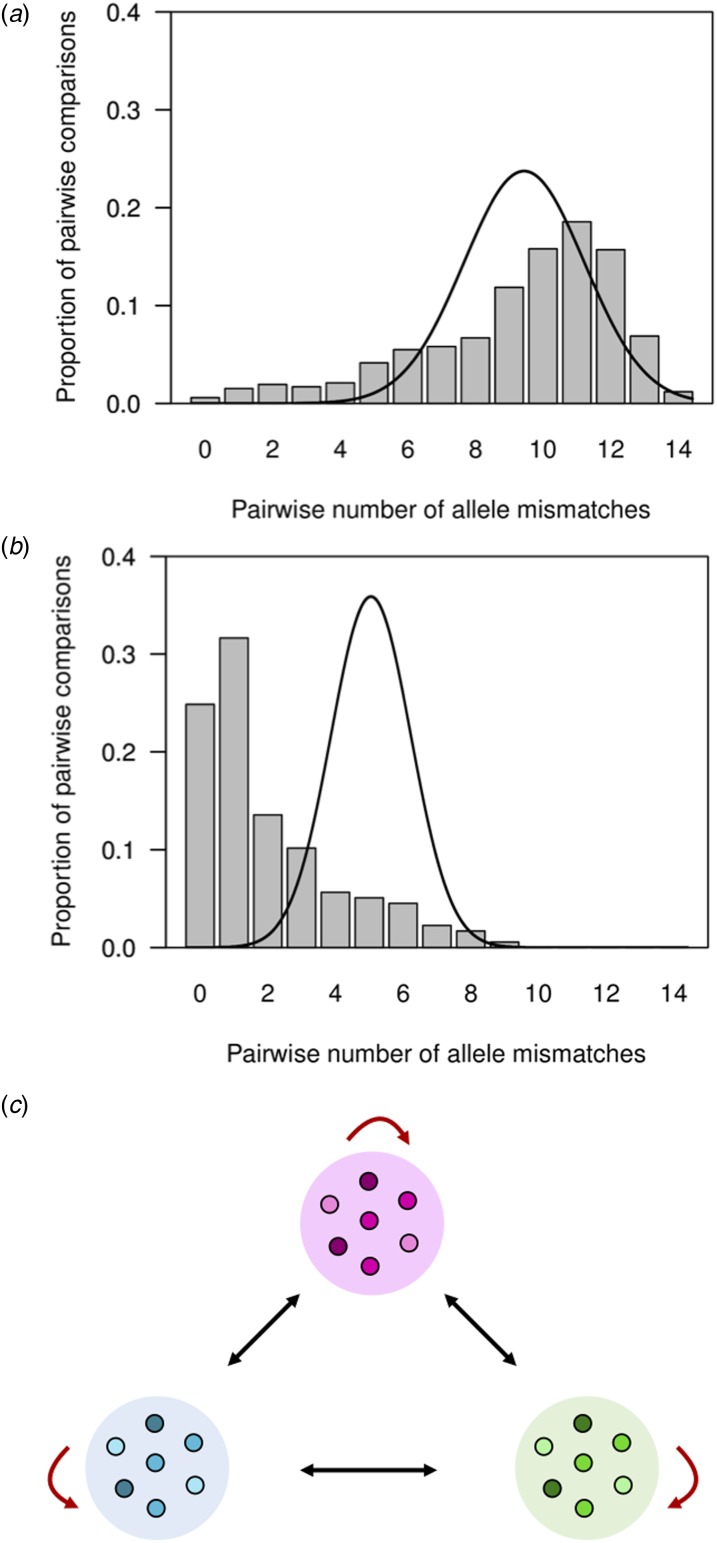
Pairwise comparisons of *P. vivax* haplotypes from independent infections in an urban setting in north-western Brazil. For each infection, the predominant allele at each of the 14 microsatellite loci analysed was considered for defining haplotypes. (a) Observed number of different alleles or mismatches in pairwise comparisons of haplotypes from 177 completely genotyped *P. vivax* isolates (bars) and expected number of mismatches under random association of alleles in a panmictic population (continuous line; data from 50 000 simulated datasets in which alleles were randomly reshuffled among haplotypes). Note that the observed (9.3) and expected (9.2) average numbers of allele mismatches were quite similar, although the overall distributions differ. (b) Observed distribution of the number of mismatches between each haplotype and its most closely related haplotype in the population, or ‘distance-to-nearest distribution’ (represented by bars), and expected distance-to-nearest distribution under random association of alleles (continuous line; data from 50 000 simulated datasets as above). The observed average distance to the nearest neighbour (2.0 mismatches) was much smaller than the expected average distance (5.1 mismatches). (c) Reasons for the departure from panmictic expectations in pairwise comparisons between haplotypes. The excess of pairwise comparisons between closely related haplotypes sharing ≥12 alleles observed in our population putatively arises from comparisons between isolates *within* clonal lineages or haplogroups (similarly coloured symbols within circles), as indicated by red arrows. Conversely, the excess of pairwise comparisons between distantly related haplotypes sharing ⩽3 alleles putatively arises from comparisons between isolates *from different clonal lineages*, as indicated by black arrows.

The distribution of allele mismatches between haplotypes (Fig. 3a) and the distance-to-nearest distribution (Fig. 3b) deviate from expectations under random assortment of alleles. Instead, they are consistent with a parasite population fragmented into clonal or near-clonal lineages of closely related haplotypes (Fig. 3c). We observe in Figure 3a an excess of pairwise comparisons between haplotypes sharing ≥12 alleles (observed, 4.3%; expected, <0.1%), arising from isolates *within* the same clonal lineages or haplogroups (see Fig. 3c). We also observe an excess of pairwise comparisons between distantly related haplotypes sharing ⩽3 alleles (observed, 43.3%; expected, 8.4%), arising from isolates *from different* haplogroups (see Fig. 3c). Furthermore, 124 (70.1%) isolates (*vs.* <0.1% expected in a randomly mating population) had their nearest haplotype ⩽2 mismatches apart (Fig. 3b).

**Fig. 4.**
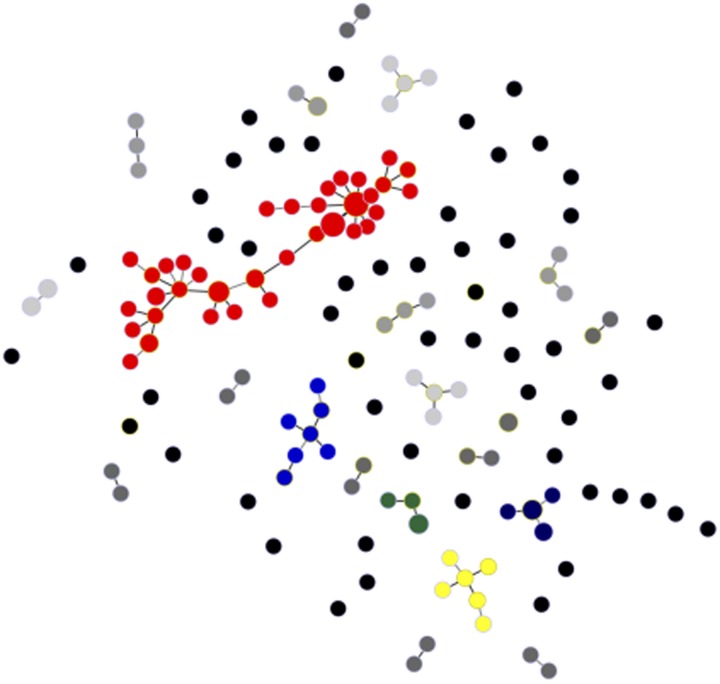
Minimal spanning tree representing the connectivity among haplotypes within 21 haplogroups found in the urban *P. vivax* population. Note that the most common of them, haplogroup 1, is represented in red and comprises 57 isolates and 35 unique haplotypes; six haplotypes within haplogroup 1 are shared by ≥2 isolates. Circles represent haplotypes with size logarithmically proportional to the number of isolates sharing them. The largest haplogroups (≥5 isolates) are colour coded as in Supplementary Fig. S1. Haplogroups with four, three and two isolates are represented with increasingly darker grey tones and singletons are shown as unconnected black circles. Lines connect pairs of haplotypes with one or two allele mismatches and the overall network represents the most likely haplotype genealogy generated by the goeBURST algorithm.

We recovered 146 unique haplotypes from 177 completely genotyped isolates; eight haplotypes were shared by a pair of isolates, two were shared by three isolates, one was shared by four isolates, two were shared by as many as nine isolates each and 133 haplotypes were recovered only once. Clustering of microsatellite haplotypes using a stringent similarity threshold (⩽2 allele mismatches) identified 21 haplogroups in the population, each comprising between 2 and 57 isolates (Fig. 4). Only 53 haplotypes (36.3% of all unique haplotypes in the population) were ≥2 alleles apart from all others and could not be assigned to any haplogroup. These ungrouped haplotypes were termed ‘singletons’ (Supplementary Fig. S1).

We found highly significant LD 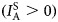 in four separate analyses (Table 2). We first considered all samples and microsatellite markers. The second analysis was done on a ‘clone-censored’ database containing only unique haplotypes, to test whether LD was due to the clonal expansion of particular haplotypes in an otherwise panmictic population [30]. We next analysed a database containing only infections with ⩽1 locus showing multiple alleles, to exclude possible errors when reconstructing predominant haplotypes in complex multiple-clone infections. Because we had markers mapping to the same chromosome (Table 1), our final analysis considered all samples but only one microsatellite marker was retained per chromosome (total of 10 microsatellite loci) to exclude physical linkage between markers along the same chromosome as a cause of LD. For this purpose, we generated 12 different databases that retained either MS2, MS4 or MS5 on chromosome 2, either MS12 or MS15 on chromosome 5 and either MS7 or MS8 on chromosome 12. We conclude that infrequent outcrossing between parasite lineages circulating in the town, rather than epidemic clonal expansions or physical linkage between markers, is the most likely cause of LD in this population.

**Table 2.** Standardised index of association 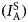 as a measure of multi-locus LD in *P. vivax* isolates from the town of Mâncio Lima, Brazil (2014–15)

Samples analysed (*n*)	No. of markers	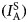	*P*^a^
All infections (177)	14	0.158	<0.0001
Unique haplotypes (146)	14	0.113	<0.0001
Single-clone infections (148)^b^	14	0.177	<0.0001
One marker per chromosome (177)^c^	10	0.160	<0.0001

a Test of the null hypothesis that 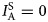.

b Only infections with ⩽1 locus showing multiple alleles were analysed, to exclude possible errors when reconstructing predominant haplotypes in multiple-clone infections.

c We generated 12 different databases comprising 10-locus haplotypes. In each haplotype, we retained either MS2, MS4 or MS5 (all on chromosome 2), either MS12 or MS15 (on chromosome 5) and either MS7 or MS8 (on chromosome 12). The 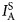 estimate is an average of all 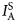 obtained with these 12 databases (all of them associated with *P* values <10^−4^).

### Near-clonal lineages across time and space

**Fig. 5.**
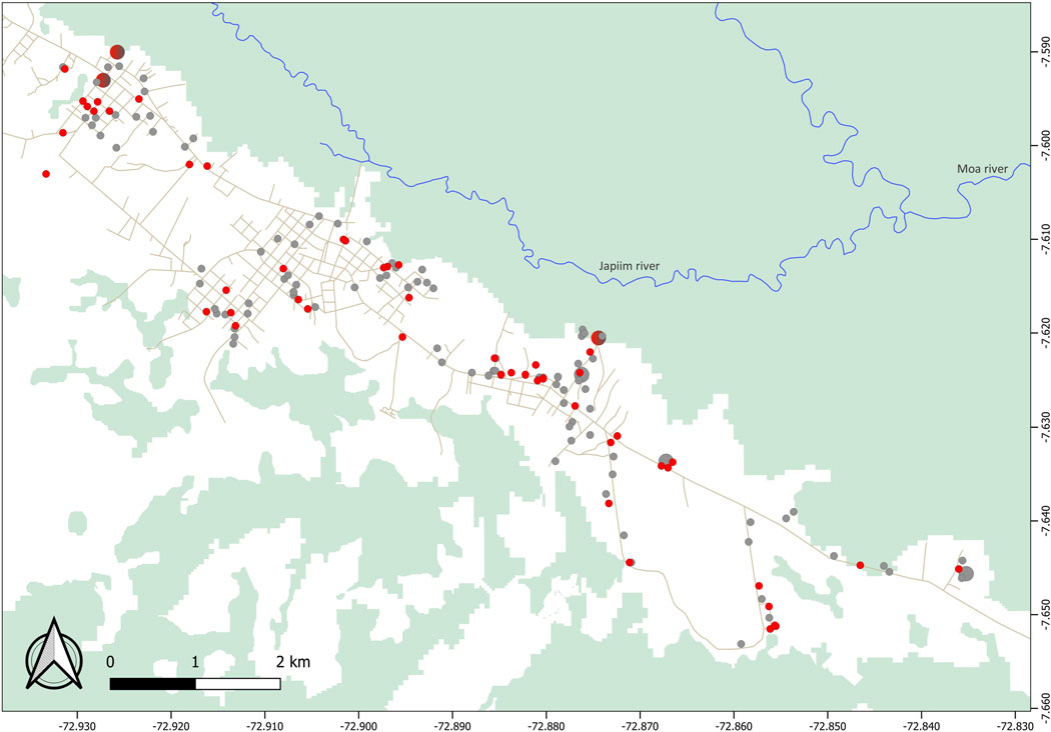
Spatial distribution of parasites within haplogroup 1 (red circles) and all other haplotypes (grey circles) found to circulate in Mâncio Lima. We show data for 155 study subjects (including 52 subjects carrying haplogroup 1 parasites); two houses with GPS coordinates fell outside the urban centre. Circle sizes are linearly proportional to the number of infections diagnosed in the same household. Note that haplogroup 1 parasites were found in patients from across the town, with no apparent spatial clustering.

The largest cluster of genetically related parasites found in Mâncio Lima (haplogroup 1) comprised 57 isolates (32.2% of all isolates genotyped) and 35 unique haplotypes (24.0% of all unique haplotypes) that are represented in red in Figure 4. Six haplotypes within haplogroup 1 were shared by ≥2 isolates (Fig. 4). This is the most likely example of endemic, locally transmitted haplogroup in this setting. Interestingly, haplogroup 1 parasites were sampled on every single month between June 2014 and June 2015 (Supplementary Fig. S1 and Movies S1 and S2); the proportion of haplogroup 1 parasites per month ranged from 71.4% (5 of 7) isolates genotyped in December 2014 to 22.2% (6 of 27) in January 2015 (Supplementary Fig. S1). This large near-clonal lineage was found in patients from across the town, with no apparent spatial clustering (Fig. 5; see also Supplementary Movies S1 and S2). These findings are very unlikely under the hypothesis of exclusively imported infections being diagnosed in urban dwellers (Fig. 1a), but are consistent with the sustained local transmission of a dominant *P. vivax* lineage over the study period. Interestingly, if urban malaria transmission does occur, visitors from the town can also introduce locally circulating parasites into neighbouring rural villages, indicating that the urban centre may be both a source and sink of parasites (Fig. 1b).

The second largest clusters, haplogroup 2 (represented in blue in Fig. 4) and haplogroup 13 (represented in dark blue in Fig. 4), comprised seven isolates each, followed by haplogroup 3 (represented in yellow in Fig. 4), with six isolates. In addition to haplogroup 1, other common haplogroups, such as haplogroup 2 and haplogroup 3, were spread over time, being recovered from infections diagnosed on ≥3 non-consecutive months. Overall, no apparent temporal clustering of relatively large haplogroups, consistent with a clonal outbreak, was observed during the 14 months of study (Supplementary Fig. S1).

## Discussion

Molecular genotyping has recently been explored to characterise malaria parasite connectivity across sites and infer potential source and sink communities [31, 32]. Understanding the source–sink dynamics is critical to determine, for example, the relative contribution of travel-related and locally acquired infections to urban [2, 10] and cross-border [31, 32] malaria burden and properly design control interventions. Here we suggest that the source–sink dynamics of urban malaria can be inferred from parasite-genotyping data even if samples from the most likely rural sources are unavailable.

We have characterised an urban *P. vivax* population that is fragmented into clonal or near-clonal lineages. Consistent with previous findings from rural sites across the Amazon Basin [29, 33, 34], only 25% parasites shared exactly the same multi-locus microsatellite haplotype with another parasite sampled from a different subject at a different time point (Fig. 4b). The continuous migration of new strains [29, 33], random genetic drift and the high mutation rates of microsatellites (about 10^−7^ per locus per asexual cycle in malaria parasites [35]) are the most likely explanations for the relatively fast haplotype replacement rate typically found in longitudinal studies of *P. vivax* populations.

We examined the genetic connectivity of parasites circulating in the urban population over 14 months. Although completely identical haplotypes were relatively infrequent in unrelated infections, 70% of isolates shared ≥12 alleles (out of 14 loci typed) with one or more samples, clearly deviating from expectations for a randomly mating population. Significantly, nearly one-third of genotyped isolates clustered into a single endemic haplogroup, which was widely distributed over space and time. This finding would be extremely unlikely in a scenario where all infections among urban residents are independent imports from different rural villages (Fig. 1a). In contrast, they are consistent with urban transmission chains of parasite lineages that gradually diversify over time (Fig. 1b). The documented occurrence of larval habitats across the town [19, 20] further indicates that urban malaria transmission can be sustained in this urban setting.

One can argue, however, that a ubiquitous parasite lineage might have been imported from multiple source villages surrounding the urban centre. The source–sink model shown in Figure 1a, instead, assumes limited gene flow between source villages and random genetic drift within each village, leading to a strong spatial structure in parasite diversity. Although we have not genotyped parasites from rural sites around Mâncio Lima, we consider very unlikely to find genetically related parasites spread across sparse, poorly connected localities. Alternatively, one might assume that closely related parasites have been imported from a single source community, e.g. from a frequently visited settlement with a homogeneous and stable parasite population, over 13 consecutive months. Our currently available travel survey data argue against the hypothesis of one or a few main rural sources of imported malaria infections. Although 30% of urban residents in Mâncio Lima report having spent at least one night in one or more rural sites within the past 6 months, no single locality with intense malaria transmission stands out as the main travel destination. In contrast, the most commonly travel destinations, mentioned by 3.5% of urban residents (I. C. Johansen, unpublished data), were riverine settlements surrounded by rainforest along the Moa and Azul rivers, where malaria incidence remains low [36, 37].

Studies of source–sink dynamics should ideally include samples from all relevant source and sink communities. Nevertheless, this and previous molecular epidemiology studies of urban malaria in South America (e.g. [11]) were restricted to parasite genotypes circulating in urban centres. An extensive sampling of parasites from rural communities over several months would represent a major logistic challenge. Indeed, rural inhabitants represent half of the total population in the municipality of Mâncio Lima and are widely dispersed into more than 50 small localities along rivers or unpaved roads, intermingled with uninhabited areas that account for 67% of the municipality's vast territory [37]. A second limitation of the current study arises from the fact that we have sampled only symptomatic infections. One can argue that the disproportionate frequency of a single parasite lineage observed in symptomatic infections may simply reflect its increased virulence, relative to all other endemic lineages that silently circulate among asymptomatic hosts in the same communities. Although our previous molecular-genotyping studies suggest that haplotype frequencies in symptomatic *P. vivax* carriers are representative of all parasites circulating in the rural Amazon [29], we recommend further investigation into this topic.

Despite these limitations, we interpret the presence of an endemic near-clonal parasite lineage circulating in symptomatic infections in the town over 13 consecutive months as a strong evidence of self-sustained urban malaria transmission in an Amazonian setting. Therefore, we suggest that interventions targeting urban dwellings are critical to eliminate malaria transmission in the main hotspot of Brazil and similar urbanised settings in South America where vector proliferation has been favoured by man-made larval habitats such as fish-farming ponds [38].

## Supplementary material

For supplementary material accompanying this paper visit https://doi.org/10.1017/S0950268820000515. click here to view supplementary material
