# Supplementary figures and images for: Molecular evidence of sustained urban malaria transmission in Amazonian Brazil, 2014–2015
Source: Epidemiol Infect. 2020 Feb 21;148:e47. doi: 10.1017/S0950268820000515 (PMC7078511; doi:10.1017/S0950268820000515)

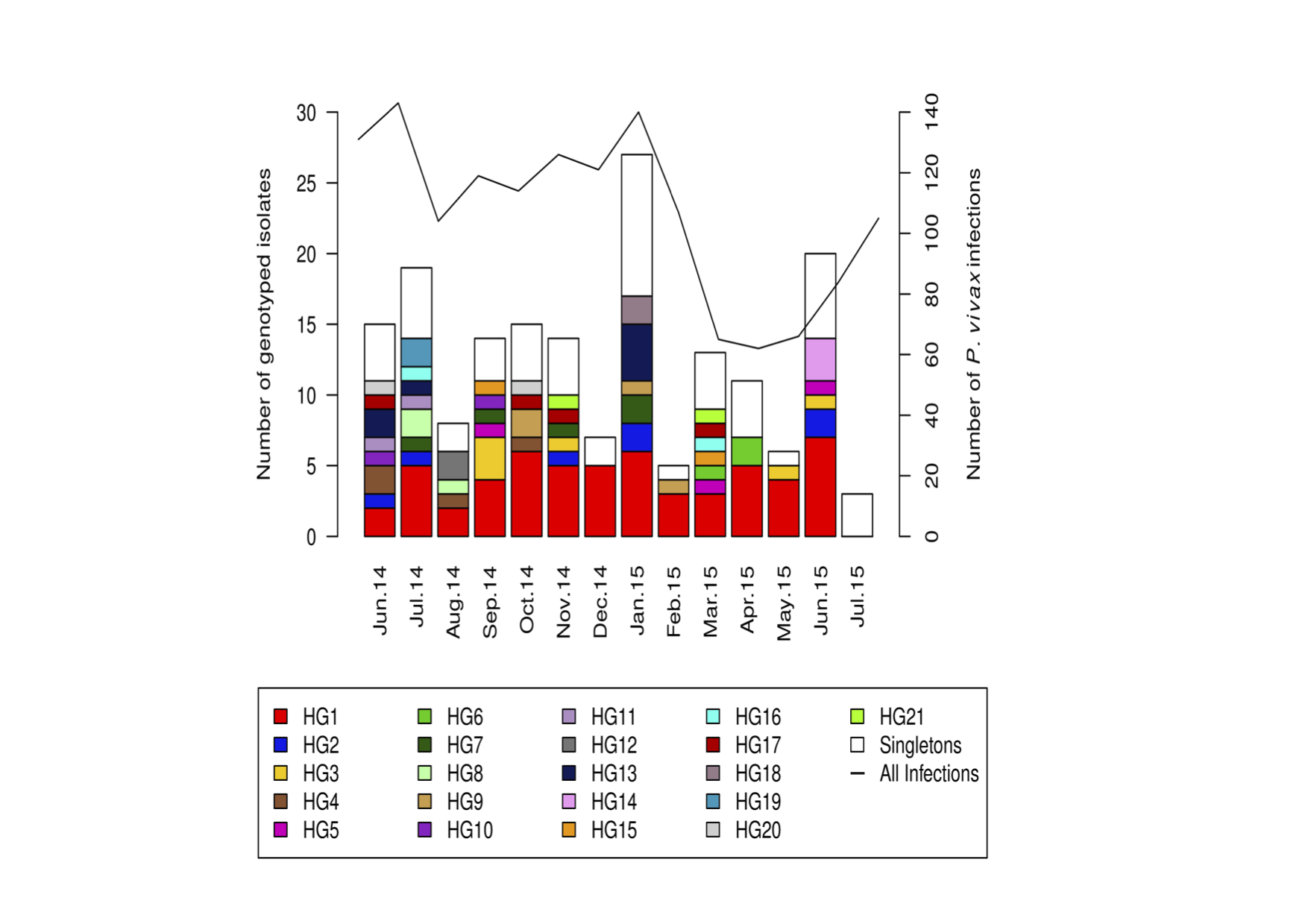

Supplement: Supplementary file 1 [file S0950268820000515sup001.zip › Figure S1.tiff]
